# A Novel Missense *MEN1* Mutation in a Sporadic Case of Multiple Endocrine Neoplasia Type 1 Complicated with Papillary Thyroid Carcinoma

**DOI:** 10.31662/jmaj.2022-0187

**Published:** 2023-03-06

**Authors:** Koji Shibuya, Ken Ebihara, Manabu Takahashi, Tomoyuki Kurashina, Shuichi Nagashima, Kenta Okada, Shun Ishibashi

**Affiliations:** 1 Division of Endocrinology and Metabolism, Department of Internal Medicine, Jichi Medical University School of Medicine, Shimotsuke, Japan

**Keywords:** MEN1, papillary thyroid carcinoma, missense mutation

## Abstract

Multiple endocrine neoplasia type 1 (MEN1) is a rare genetic disorder, resulting from *MEN1* gene abnormalities, which causes tumors mainly in the endocrine glands. We experienced a sporadic case of MEN1 complicated with papillary thyroid carcinoma (PTC) and found a novel missense mutation in the patient’s *MEN1* gene. Her older sister, who showed no typical symptom of MEN1, had a history of PTC, suggesting the presence of another genetic factor involved in PTC development. This case suggests the importance of an individual’s genetic background in the development of MEN1 complications.

## Introduction

Multiple endocrine neoplasia type 1 (MEN1) is a rare genetic disorder that causes tumors mainly in the endocrine glands, including the parathyroid glands, the pancreas, and the pituitary gland. MEN1 can also cause tumors in other endocrine glands, such as the adrenal glands and the thyroid gland. MEN1 is an autosomal dominant disorder caused by *MEN1* gene abnormalities. However, about 1 in 10 cases of MEN1 are sporadic. We herein report a sporadic case of MEN1 complicated with papillary thyroid carcinoma (PTC) and a novel missense mutation in the patient’s *MEN1* gene.

## Case Report

**Figure 1.**
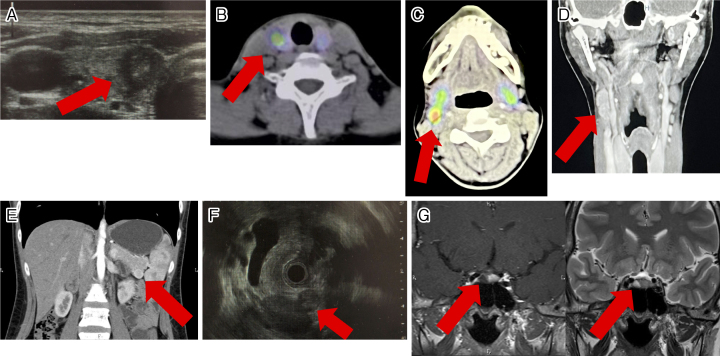
Diagnosis of thyroid tumor and MEN1 by imaging. (A) Ultrasonography of the thyroid gland. (B) ^99m^Tc-MIBI scintigraphy of parathyroid glands. (C) ^99m^Tc-MIBI scintigraphy of the submental region. (D) CT scan of the neck. (E) CT scan of the pancreas. (F) Endoscopic ultrasonography of the pancreas. (G) Enhanced MRI of the pituitary gland. T1 and T2 weighted images (left and right, respectively). Red arrows indicate sites of lesions.

A 29-year-old Japanese woman presented to our department for hypercalcemia. She had an isthmectomy for a median adenomatous goiter 5 years ago. This time, thyroid masses were pointed out again at her workplace health examination. Ultrasonography confirmed a 7-mm mass with border irregularity and sand grain calcification, which was diagnosed as PTC by fine needle aspiration cytology (Figure 1A). This diagnosis was later confirmed in a resection specimen. As hypercalcemia at the level of 12.5 mg/dL was found by a preoperative blood test, she was referred to our department for further examination.

Her intact-parathyroid hormone level was obviously elevated (Table 1). Although her FECa value was low, this was thought to be due to vitamin D deficiency (Table 1) ^(1)^. ^99m^Tc-MIBI scintigraphy showed abnormal accumulations in the lower right parathyroid gland and the right submental region (Figure 1B and 1C). Although no parathyroid gland swelling was detected with thyroid ultrasonography, a cervical computed tomography (CT) scan showed a tumor near the right carotid sinus (Figure 1D). Pathology with later surgical resection specimens revealed that the cervical tumor was an ectopic parathyroid adenoma and the lower right parathyroid gland was normal tissue. The cervical tumor was considered to be the lesion responsible for hyperparathyroidism. We conducted screening tests for MEN1. An abdominal CT scan showed a tumor in the pancreas, which was revealed as a pancreatic neuroendocrine tumor (P-NET) by endoscopic ultrasound-fine needle aspiration cytology (Figure 1E and 1F). Magnetic resonance imaging (MRI) of the head revealed a pituitary tumor suspected to be an adenoma (Figure 1G). Based on hormonal testing, both her P-NET and pituitary tumor were considered nonfunctional. Given the above results, the patient was clinically diagnosed with MEN1. At her request, right lobe thyroidectomy and cervical tumor resection were performed.

**Table 1.** The Patient’s Clinical Findings and Laboratory Data on an Initial Medical Examination.

	This case	Normal range
SBP/DBP (mmHg)	106/68	130/80
BMI (kg/m^2^)	19.3	18.5-25
FPG (mg/dL)	85	73-109
ALP (IU/L)	695	106-322
Na (mmol/L)	139	138-145
K (mmol/L)	4.4	3.6-4.8
Ca (mg/dL)	12.5	8.8-10.1
P (mg/dL)	2.2	2.7-4.6
Mg (mg/dL)	2.3	1.7-2.5
FECa (%)	0.67	<1.0
Intact-PTH (pg/mL)	496	10-65
ACTH (pg/mL)	52.1	7.2-63.3
F (μg/dL)	12.1	6.24-18.0
TSH (μU/mL)	1.17	0.45-3.33
FT3 (pg/mL)	2.37	2.11-3.51
FT4 (ng/dL)	1.09	0.84-1.44
GH (ng/mL)	0.7	<2.1
IGF-1 (ng/mL)	188	133-312
PRL (ng/mL)	11.3	3.12-15.39
LH (mIU/mL)	7.6	0.5-68.7
FSH (mIU/mL)	7.3	1.5-20.6
C-peptide (ng/mL)	1.51	1.1-3.3
Insulin (μU/mL)	7.9	<16
Gastrin (pg/mL)	95	<200
Glucagon (pg/mL)	208	70-174
Thyroglobulin (ng/mL)	106	<33.7
25-hydroxyvitamin D (ng/mL)	9.9	>20

We performed gene analysis on the *MEN1* gene. Direct sequencing of the coding region disclosed a novel heterozygous G to C mutation at nucleotide 293 in exon 2 (Table 2). This mutation predicts the substitution of arginine by proline at codon 98 (R98P). We could not find any reports of the same mutation but found two mutations with different substitutions at the same site in databases available on the internet. Although there was no information on the clinical significance of one mutation, R98Q reported in ClinVar, the carrier of the other mutation, R98L was diagnosed with MEN1 clinically (Table 2) ^(2)^. Furthermore, both analytical tools, Polyphen2 and PROVEAN, predicted that the R98P mutation had a damaging or deleterious impact on the biological function of the MEN1 protein (Table 2). Taken together, we concluded that the R98P mutation was the cause of her MEN1 phenotype. Although we did not perform gene analysis in other family members, none of them showed any typical symptoms of MEN1 including hypercalcemia, although one of her older sisters had a history of thyroidectomy for PTC at the age of 34 years. Thus, she was considered a sporadic case for MEN1 and her *MEN1* mutation was *de novo*.

**Table 2.** Reported Substitutions of Arginine at Codon 98 of the MEN1 Protein and Their Predictions of Functional Effects.

Nucleotide change (amino acid change)	Source	PolyPhen2 (Score/prediction)	PROVEAN (Score/prediction)
c.293G>C (p.R98P)	This case	0.995/probably damaging	-4.805/deleterious
c.293G>A (p.R98Q)	ClinVar	0.743/possibly damaging	-2.341/neutral
c.293G>T (p.R98L)	Article (1)	0.966/probably damaging	-4.889/deleterious

## Discussion

We reported a sporadic case of MEN1 complicated with PTC. MEN1 mutation can cause thyroid tumors. The complication rates of thyroid tumors with MEN1 vary in reports, ranging from 2.6% to 25% ^(3)^. When limited to PTC, the complication rate was reported to be 4.7% in one study ^(4)^. On the other hand, one of the older sisters of this patient who showed no typical symptoms of MEN1 had a history of PTC, suggesting the presence of another genetic factor involved in the development of PTC; however, the involvement of the *MEN1* mutation in the pathogenesis of PTC cannot be denied.

We also reported a novel missense mutation in exon2 of the *MEN1* gene. This might be a *de novo* mutation as this case was sporadic. *MEN1* mutations reported so far are distributed across the entire coding region, including exon2 without a hot spot ^(5)^. In contrast to MEN2, MEN1 has no obvious genotype-phenotype relationship ^(6)^. MEN1 symptoms can differ from person to person, even among family members who have the same *MEN1* mutation. These facts indicate that an individual’s genetic background plays an important role in the development of MEN1 complications. In this case, genetic factors involved in the pathogenesis of PTC in the patient and her older sister might affect her MEN1 complications.

In conclusion, we experienced a sporadic case of MEN1 complicated with PTC and found a novel missense mutation in the patient’s *MEN1* gene. This case suggests the importance of an individual’s genetic background in the development of MEN1 complications.

## Article Information

### Conflicts of Interest

None

### Author Contributions

KS was involved in the evaluation and management of the patient. KS and KE did the literature search and drafted the manuscript. MT, TK, SN, KO, and SI revised the manuscript critically. All the authors declare that they contributed to this article and that they read and approved the final version.

### Informed Consent

The patient signed informed consent regarding publishing this case in an academic journal.
